# Genomic insights from *Paraclostridium bifermentans* HD0315_2: General features and pathogenic potential

**DOI:** 10.3389/fmicb.2022.928153

**Published:** 2022-08-24

**Authors:** Hailan Zhao, Jiaqi Wang, Yao Peng, Xunchao Cai, Yandi Liu, Wenqi Huang, Hongli Huang, Yuqiang Nie

**Affiliations:** ^1^Department of Gastroenterology and Hepatology, The Second Affiliated Hospital, School of Medicine, South China University of Technology, Guangzhou, Guangdong, China; ^2^Department of Gastroenterology and Hepatology, Guangzhou First People's Hospital, School of Medicine, South China University of Technology, Guangzhou, Guangdong, China; ^3^Department of Gastroenterology and Hepatology, Shenzhen University General Hospital, Shenzhen, Guangdong, China

**Keywords:** *Paraclostridium*, *P. bifermentans*, antimicrobial resistance, pathogenicity, *Listeria* LIPI-1, multiple plasmids, phylogenomic tree

## Abstract

**Background:**

*Paraclostridium bifermentans* is the most diverse distributed species of *Paraclostridium* and can cause fatal human infections under rare conditions. However, its pathogenic mechanisms and adaptation ability behind infections remain unclear. Herein, we reported the complete genome sequence of *P. bifermentans* HD0315_2 isolated from the feces of a patient with Crohn's disease. Then, we performed genomic analyses to understand its pathogenic mechanisms and adaptation ability.

**Results:**

The *de novo* assembly revealed that the HD0315_2 strain carried a circular chromosome of 3.27 Mb and six circular plasmids (19.41 to 139.50 kb). The phylogenomic analysis assigned the HD0315_2 strain as *P. bifermentans* and reclassified some previously non-*P. bifermentans* strains into this clade. The general genomic features showed that this species harbored a flexible genomic pool characterized by variable genome length and multiple plasmids. Then, the HD0315_2 strain was predicted as a human pathogen with high probability, and *Listeria* LIPI-1 virulence proteins were identified on its genome. Besides, abundant antibiotics/metal/stress resistant genes, such as *asrABCH, cat, mccF, macB, entS, albA, bcrA*, and *tetB*, were carried by either the genome or the plasmids. Furthermore, we proposed that transposase-directed horizontal gene transfer was responsible for the distribution of multiple copies of the *hin* gene in the plasmids.

**Conclusion:**

The flexible genomic pool of *P. bifermentans* encodes abundant functions for antimicrobial or oxidative stress resistance, helping it successfully inhabit and adapt to diverse environments. Moreover, *P. bifermentans* HD0315_2 might infect hosts *via* a *Listeria* LIPI-1-like cycle, with the help of a plasmid expressing the Hin DNA invertase to evade host immune responses.

## Introduction

*Paraclostridium* spp. belongs to the class Clostridia of Gram-positive, rod-shaped, and obligate anaerobe bacteria that reside in various mesophilic conditions, such as soil, marine habitats, polluted waters, and clinical specimens (Jyothsna et al., 2016). To date, only two species have been formally published under this genus (i.e., *P. bifermentans* and *P. benzoelyticum*) (Jyothsna et al., 2016). Among them, the most frequently isolated and best-characterized species is *P. bifermentans* (Hale et al., 2016). Additionally, in 2016, *P. bifermentans* was reclassified from *Clostridium bifermentans* (Jyothsna et al., 2016). This species has been previously recognized as nonpathogenic unless co-inhabited with *Clostridium perfringens*, producing putrid lesions in guinea pigs (Weinberg and Séguin, 1918). However, 15 cases of *P. bifermentans* infections in humans have been recently summarized and included brain abscess, lymphadenitis, necrotizing endometritis, a prosthetic knee joint infection, empyema, and endocarditis (Kolander et al., 1989; Edagiz et al., 2015; Hale et al., 2016; Biswas et al., 2018; Barrett et al., 2020). Kutsuna et al. have shown that *P. bifermentans* PAGU1678^T^ exacerbates mouse's pathological conditions in a dextran sulfate sodium-induced colitis model (Kutsuna et al., 2018). Although *P. bifermentans* has emerged as a rare pathogen in humans, it thrives as a human pathogen under specific conditions and rises to major health hazards. Nevertheless, no report has determined any suggestive genes causing its pathogenicity, although various draft genomes have been published. On the other hand, the high similarity of 16S rRNA sequences within the genus (*e.g., P. bifermentans* and *P. benzoelyticum* share a 16S rRNA sequence similarity > 99%) is difficult for their taxonomic identification and characterization of biological traits. Herein, we isolated *P. bifermentans* strains from the feces of patients with Crohn's disease (CD) for the first time. Additionally, we showed that *P. bifermentans* strains were only isolated from patients with CD but not from patients with ulcerative colitis (UC), underlying the potential involvement of this species in CD. Then, the complete genome of *P. bifermentans* HD0315_2, isolated from the feces of a young male patient with CD, was assembled *de novo* using reads from Illumina and Nanopore sequencing. Furthermore, whole-genome sequence-based taxonomy identification and comparative genome analyses were performed to clarify the general genome function and its specificities regarding virulence, adaptation, and pathogenic effects.

## Materials and methods

### Isolation and characterization of strains

Anaerobic conditions (90% N_2_, 5% CO_2_, and 5% H_2_) were used during the isolation and culture of *P. bifermentans* strains. First, fresh fecal samples were collected from a 24-year-old Chinese man with active CD from Guangdong (China) who suffered from recurrent abdominal pain, changes in stool traits, and perianal exudate. Briefly, 10 ml of sterile PBS was added per gram of fresh feces to a 50-ml conical tube, then vortexed and left to settle. Next, the feces suspension was transferred to anaerobic blood culture bottles (BD, BACTECTM Lytic/10 Anaerobic/F Culture Vials, America) supplemented with sterile sheep blood and rumen fluid. Bottles were incubated under anaerobic conditions at 37°C for 30 d as described by Lagier's culturomics strategy (Lagier et al., 2018). Then, 1 ml of suspension was sterilely aspirated from the incubated culture and serially diluted (10 to 10^12^). Finally, 100 μl of each dilution was evenly plated on Brain-Heart Infusion (BHI) or reinforced clostridia agar plates to harvest colonies. Purification was further conducted by streaking. The harvested colonies were enriched in BHI medium at 37°C for 3 d and identified by MALDI Biotyper RTC (Bruker Daltonics, Germany). Single and sufficiently grown colonies were directly transferred to the MALDI Biotyper RTC 96 target spot, and 1 μl of Bruker bacterial test standard (BTS) and matrix solution were sequentially added to prepare the detection target. A spectrum score of 2.3 was used as the threshold for high-confident taxa identification at the species level. Then, the 16S rRNA sequence of one strain (HD0315_2), identified as *Paraclostridium* sp., was obtained by PCR using the 8f/1492r primer pair and sent to Beijing Genomics institution (BGI) for Sanger sequencing. For taxa identification, the 16S rRNA sequence was aligned using the NCBI nucleotide (nt) collection database. Finally, species were determined with 100% sequence coverage and > 97% sequence identity.

### Genome sequencing and assembly

Genomic DNA extraction was performed using the TaKaRa MiniBEST Bacteria Genomic DNA Extraction Kit (Takara, Japan) following the manufacturer's instructions. The DNA quality was checked using the Synergy HTX Multi-Mode Reader (BioTek, USA). Genome sequencing was separately performed under two platforms to generate long and short reads by the Nanopore PromethION platform (MAGIGENE, Guangzhou, China) and the Illumina NovaSeq platform (Novogene, Nanjing, China), respectively. The long-read sequencing library was constructed using the SQK-LSK109 kit (Oxford Nanopore Technologies, UK) according to the manufacturer's instructions. Sequencing and base calling were performed using MinKNOW v1.15.4 with the FLO-MINSP6 flow cell (Oxford Nanopore Technologies, UK) and low-quality reads (i.e., scores <7) were removed. The short-read sequencing library was constructed with a 350 bp insert size and sequenced using the PE150 strategy. Adapter trimming and low-quality reads (Phred score ≤ 20) were removed for quality control. The *de novo* genome assembly was conducted using the Unicycler v0.4.9b assembler (Wick et al., 2017), with the default hybrid assembly pipeline.

### Phylogenomic characterization and plasmid detection

Taxonomy assignment was further curated using the gtdbtk_wf workflow implemented in GTDT-Tk (Parks et al., 2018). The average nucleotide identity (ANI) between the HD0315_2 strain and the phylogenomic close genomes were calculated using fastANI (Jain et al., 2018). A phylogenomic tree based on the whole genome protein sequences was constructed using CVTree3 with default parameters (K-tuple length 3, 4, 5, 6, 7) (Zuo and Hao, 2015). Plasmids were predicted using PlasForest v1.2 (https://github.com/leaemiliepradier/PlasForest) and mlplasmids v2.1.0 (https://gitlab.com/sirarredondo/analysis_mlplasmids) based on machine learning from sequence homology and pentamer frequencies.

### Comprehensive genome annotation

Comprehensive genome annotation was performed using the NCBI Prokaryotic Genome Annotation Pipeline (PGAP) (Tatusova et al., 2016). Functional genome annotation with multiple databases, including Carbohydrate-Active enZYmes (CAZy), Cluster of Orthologous Groups (COG), and Kyoto Encyclopedia of Genes and Genomes (KEGG), was performed using eggNOGMapper v2.1.5 (Huerta-Cepas et al., 2017). Comprehensive genome analysis, including the annotation of subsystems, identification of specialty genes (transporters, virulence factors, drug targets, antibiotic resistance genes, and antimicrobial resistance genes), and phylogenetic analysis, was conducted using PATRIC v3.6.10 (https://www.patricbrc.org/). Pathogenicity prediction was performed using PathogenFinder v1.1 (https://cge.cbs.dtu.dk/services/PathogenFinder/). Genomic islands (GI) were identified using IslandViewer 4 (Bertelli et al., 2017).

### Quality assurance

The HD0315_2 strain was repeatedly sub-cultured on broth agar plates to confirm the obtainment of a single colony before sequencing. Taxa assignments were cross-validated by both MALDI Biotyper RTC and full-length 16S rRNA sequencing, which determined that the HD0315_2 strain belongs to the *Paraclostridium* genus (Supplementary Tables S1, S2). Quality control was conducted by using CheckM v1.0.12 to evaluate the assembly quality and using samtools v1.9 and bcftools v1.10.2 to check the variants through mapping the Illumina reads to the contigs. Then, the assembled genome sequence was loaded onto GTDB-Tk for taxonomy assignments.

## Results and discussion

### Genomic features and phylogenomic tree

The hybrid assembly using Nanopore and Illumina reads generated seven complete circular contigs without Ns. The results showed that the (1) high-quality contigs were assembled using the Unicycler assembler (contamination = 0.70%, completeness = 99.30%, Strain heterogeneity = 0.00%), (2) the pair-end Illumina reads showed an overall 99.61% alignment rate to the assembled contigs, and (3) only three-point sites were determined as SNP sites, all located in the chromosome (i.e., contig HD0315_2) (Supplementary Table S3). The plasmid identification analysis showed that the biggest contig was the chromosome sequence, while the remaining six contigs were plasmids. The taxa identification workflow implemented in GTDB-Tk identified the HD0315_2 strain as *P. bifermentans* based on the sequence ANI (> 96) from the biggest contig (chromosome). The six smaller contigs did not return the bacteria marker genes when loaded into the workflow, which validated them as plasmids. The whole-genome sequencing of *P. bifermentans* HD0315_2 comprehends a circular chromosome with 3,265,124 bp, 3, 366 CDSs, 51 rRNA, 103 tRNAs, and a G + C content of 28.8%; and six plasmids with lengths, G + C contents, and CDSs of the six plasmids are between 19,414–139,499 bp, 24.6–27.6%, and 23–162, respectively (Table 1). Currently, only two complete genomes of the *Paraclostridium* genus have been released in the NCBI genome database, and both belong to *P. bifermentans* species. Similar to the HD0315_2 strain, these two genomes carry multiple plasmids and close genome G + C content (<0.5% variations). Moreover, the chromosome lengths in this species varied from 3.27 to 3.64 Mb, indicating a flexible genomic pool not only from plasmid sequences but also from genome accessory sequences. Although the chromosome length of the HD0315_2 strain is the shortest and has a lower G/G+P proportion, it carries more rRNAs/tRNAs and is associated with more plasmids and might represent a more efficient protein synthesis capability of HD0315_2, in contrast to the Cbm strain that is characterized by an opposite pattern. Detailed comparisons of the genomic features between these strains are listed in Table 1.

**Table 1:** Genomic features of the strains in the genus *Paraclostridium* with completed genome sequences.

**Strain**	**Accession**	**Type**	**Length (bp)**	**GC%**	**CDS**	**rRNA**	**tRNA**	**Other RNA**	**Total Length (bp)**	**G/G+P (%)^a^**
*P. bifermentans* HD0315_2	CP094933	Chromosome	3, 265, 124	28.8	3, 366	51	103	4	3, 639, 934	89.7
pHD0315_2-1	CP094934	Plasmid	139, 499	26.3	162	-	-	-		
pHD0315_2-2	CP094935	Plasmid	109, 009	26.8	140	-	-	-		
pHD0315_2-3	CP094936	Plasmid	49, 869	24.6	58	-	-	-		
pHD0315_2-4	CP094937	Plasmid	32, 029	25.8	39	-	-	-		
pHD0315_2-5	CP094938	Plasmid	24, 990	27.6	34	-	-	-		
pHD0315_2-6	CP094939	Plasmid	19, 414	27.2	23	-	-	-		
*P. bifermentans* DSM14991	CP079737	Chromosome	3, 304, 778	28.8	3, 346	48	102	4	3, 566, 216	92.7
Unnamed 1	CP079738	Plasmid	142, 041	26.6	162	-	-	-		
Unnamed 2	CP079739	Plasmid	52, 569	26.5	76	-	-	-		
Unnamed 3	CP079740	Plasmid	47, 028	24.2	48	-	-	-		
Unnamed 4	CP079741	Plasmid	19, 800	26.8	21	-	-	-		
*P. bifermentans* Cbm	CP032452	Chromosome	3, 638, 572	28.5	3,486	28	52	3	3, 770, 185	96.5
pPbmMP	CP032455	Plasmid	26.1	101		-	-	-		
pPbm2_2	CP032454	Plasmid	26.4	5		-	-	-		
pPbm14_8	CP032453	Plasmid	34.4	12		-	-	-		

^a^G/G+P represents the chromosome length proportion to the whole genomic length containing the chromosome and plasmids.

**Figure 1:**
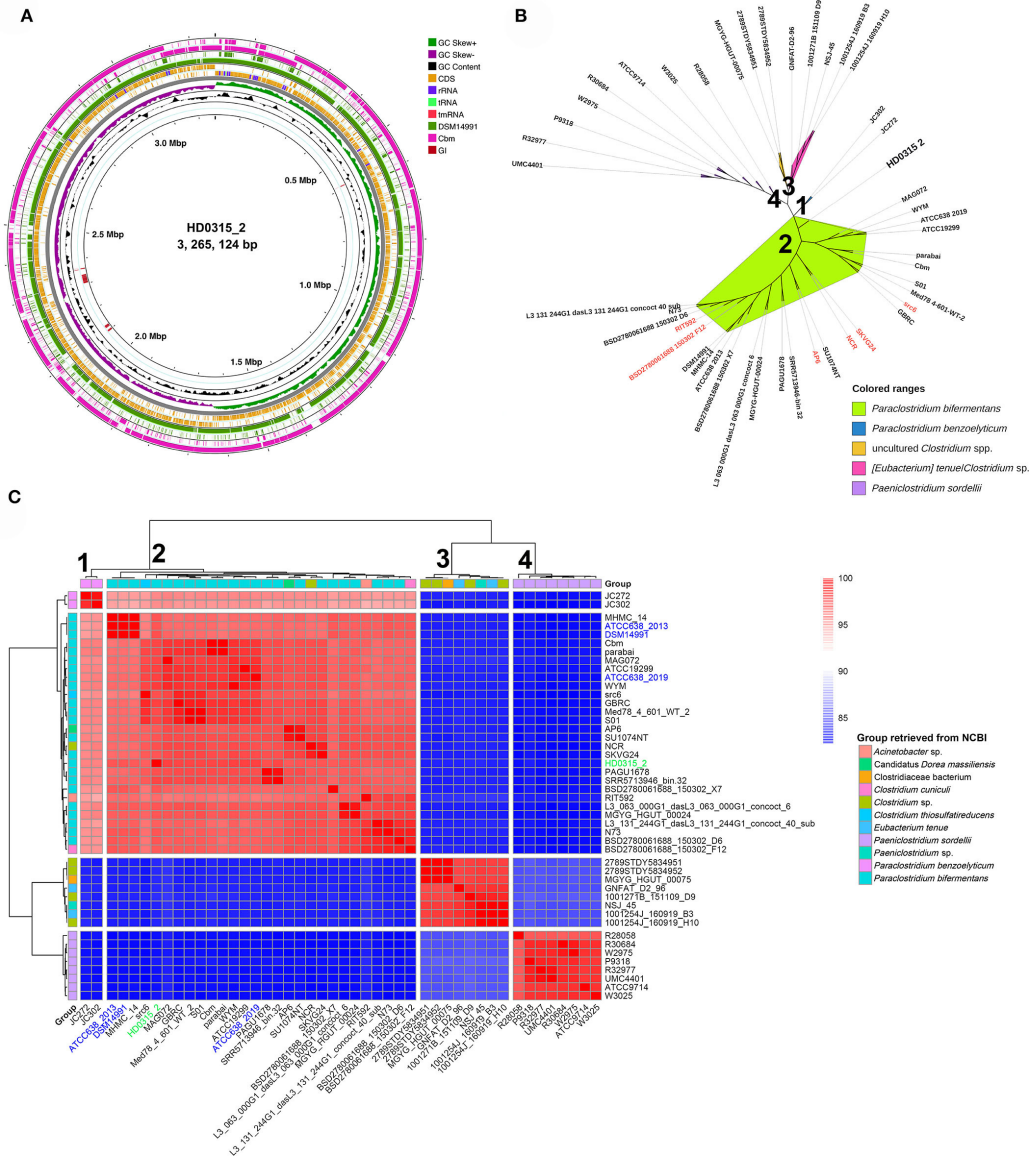
The circular display of the complete genome sequence feature and phylogenomic tree originated from *P. bifermentans* HD0315_2. **(A)** From outer to inner rings: genome sequence of *P. bifermentans* Cbm, the genome sequence of *P. bifermentans* DSM14991, CDSs on the forward/reverse strand, GC skew, GC content, and genome islands. RNAs and repeat sequence regions are displayed in the CDSs circles. GI represents genome islands. **(B)** Phylogenomic tree constructed from WGS of the phylogenetic close strains. Numbers marked on the tree branch represent four Clades divided by phylogenomic analyses. Leaf labels with red color are those reclassified as *P. bifermentans* members in this study. Previously documented taxonomic information of them are listed in Supplementary Table S1. **(C)** Heatmap displaying the ANI between the phylogenetically close *Paraclostridium* spp. The color bar on the right indicates the ANI value calculated using fastANI. Numbers marked alongside the dendrogram branch represent four Clades divided by clustering. Strains names marked with blue color are type strains of *P. bifermentans* those with different genome versions or from different institution collections (e.g., ATCC or DSM). Strain HD0315_2 is marked with green color.

The genome sequence alignment among HD0315_2, DSM14991, and Cbm strains indicated that large sequence rearrangement/transversion events happened in Cbm, close to the homologous replicon origin region of HD0315_2, which might result in the variations of rRNAs and tRNAs gain or loss (Figure 1A). Five GIs were predicted in the genome sequence, two were exclusive for HD0315_2 (located at 2,096,711–2,102,952 bp and 2,112,391–2,122,603 bp). However, none of them were assigned as pathogenic or antibiotic-resistant GIs by the IslandViewer tool. Then, we used PATRIC to collect and analyze the top 50 genomes close to HD0315_2 (indicated by the genome distance) (Supplementary Table S4). A phylogenomic tree was constructed using these genomes and showed that these strains can be divided into four major clades. The biggest clade (clade 1) consists of 28 strains that could be assigned as *P. bifermentans*. We found that some strains in clade 1 were previously assigned as non-*Paraclostridium*, such as *Clostridium* sp. NCR, Candidatus *Dorea massiliensis* AP6, *Clostridium cuniculi* BSD2780061688_150302_F12, *Clostridium thiosulfatireducens* src6, and *Acinetobacter* sp. RIT592, and reclassified as *P. bifermentans* in the present study. Moreover, SKVG24, previously proposed as the *P. dentum* type strain (Choksket et al., 2020), is proven to be an invalidly assigned species (https://lpsn.dsmz.de/search?word=Paraclostridium), was clustered into clade 1 and should be reclassified as *P. bifermentans* (Figure 1). Additionally, clade 2 was composed of *P. benzoelyticum*, which was the most phylogenomically close species to *P. bifermentans*. Clades 3 and 4 consisted of species distant from *P. bifermentans*, such as *Eubacterium tenue* and *Clostridium sordellii* (Figure 1B). A novel *P. bifermentans* subspecies, named *Paraclostridium bifermentans* subsp. *muricolitidis* subsp. nov., was recently proposed by Kutsuna et al. (2019). We found that the HD0315_2 strain was close to *Paraclostridium bifermentans* subsp. *bifermentans* (Figure 1B). The phylogenomic relationship between these strains was further validated by the ANI analysis (Figure 1C).

### General gene functions

A total of 1,188 subsystem features were revealed by RAST in the genome of *P. bifermentans* HD0315_2 (Supplementary Figure S2). Most features were assigned to “Protein Metabolism (237)” and “Amino Acids and Derivatives (179).” The wide distribution or habitats of *Paraclostridium* spp. have suggested that this genus has an excellent ability to survive in various environments (Rai et al., 2015), which was endowed by the genomic function. We found that 75 genes were tightly correlated to the adaptation of HD0315_2, including 42 assigned to “Virulence, Disease and Defense,” 7 assigned to “Phages, Prophages, Transposable elements, Plasmids,” and 26 assigned to “Stress Response” (Supplementary Figure S2). The main resistance coding genes were those sub-assigned to tetracycline (4), fluoroquinolones (2), cobalt-zinc-cadmium (11), copper (2), and multidrug-compounds (12). The virulence factor coding genes were mainly sub-assigned to *Mycobacterium-*involved virulence operon (10) and *Listeria* LIPI-1-extended proteins (3). Stress-response coding genes were mainly sub-assigned to osmotic stress (4) and oxidative stress (15) (Supplementary Table S5). However, the plasmid annotation using RAST presented poor results, and only 15 genes in the plasmids were assigned to the subsystem (data not shown). Among them, two genes were in pHD0315_2-1 code bile hydrolysis (1) and cobalt-zinc-cadmium resistance genes (1). *Paraclostridium* strains have been observed expressing multiple antibiotic-resistance genes (e.g., chloramphenicol, tetracycline, and gentamicin) (Liang et al., 2020), metal/metalloid (e.g., Se, Hg, As, and Zn) (Wang et al., 2021; Zhang et al., 2022) or toxic compounds (e.g., ciprofloxacin) (Fang et al., 2021) and were also deduced from the genomic coding functions in this study. Importantly, coding genes identified as *Listeria* LIPI-1-extended proteins in the HD0315_2 genome have been well-characterized for the infection cycle processes of “Internalization,” “Escape from the vacuole,” and “Reinfection” (see Supplementary Figure S3 for details). Overall, the functions encoded by the HD0315_2 genome contribute to survival in diverse environments. Meanwhile, for the multiple plasmids, the identification of functions was difficult due to the poor annotations.

### Pathogenicity and survival features

**Figure 2:**
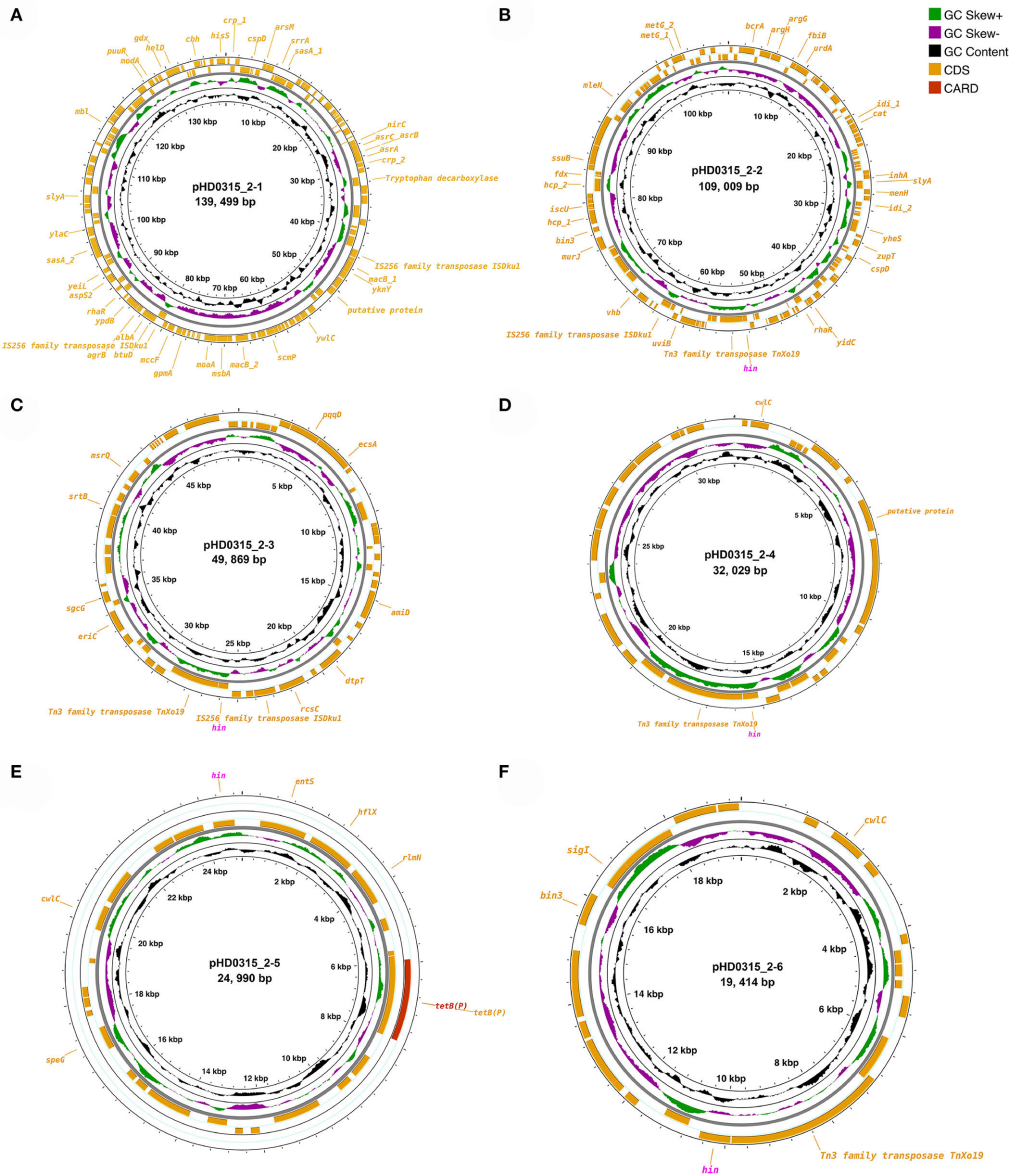
Circular display of the plasmids identified in *P. bifermentans* HD0315_2. **(A–F)** From outer to inner rings: antibiotic-resistant gene based on CARD annotation, CDSs on the forward/reverse strand, GC skew, and GC content. CDSs labeled with magenta color are genes (*hin*) coding Hin DNA invertase. CDSs without a label are genes coding hypothetical proteins.

Since various pathogenicity or virulence factor coding genes were annotated in the genome sequence, we further checked the possibility of the HD0315_2 strain being a human pathogen using PathogenFinder. The results have suggested that the HD0315_2 strain is a human pathogen with a high probability (0.77 and 0.98), besides 11 and one pathogenic protein family detected in the chromosome of HD0315_2 and the pHD0315_2-3 plasmid sequence, respectively (Supplementary Table S6). Interestingly, all pathogenic proteins detected in the chromosome sequence were homologs [e.g., putative malate-2H(+)/Na(+)-lactate antiporter, PTS-system-phospho carrier protein, and ribonuclease Ph] of the *Clostridium difficile*, and the pathogenic protein detected in the pHD0315_2-3 sequence was an amino acid/peptide transporter homolog of *Clostridium botulinum* (see Supplementary Table S4 for details). However, well-characterized toxins (e.g., enterotoxins and cytotoxins) that can cause diarrhea and inflammation by *C. difficile* (Butler et al., 2016) were not detected in the HD0315_2 strain. The detected pathogenic proteins were related to pathogen virulence and surviving regulation. For example, ribonuclease Ph is present in many pathogen ns and has been proven to help them withstand oxidative stress or oxidative bursts of neutrophils and/or macrophages from hosts (Lawal et al., 2011). Moreover, the Hin DNA invertase coding gene (i.e., *hin*) was carried by five out of the six plasmids (Figure 2). The Hin DNA invertase has been found to regulate flagellar phase variations in pathogens (Merickel and Johnson, 2001), which would help the HD0315_2 strain to evade host immune responses. Other features, not in the RAST subsystem but annotated by PGAP, included antimicrobial/metal resistance genes (e.g., *asrABCH, cat, mccF, macB, entS, albA, bcrA*, and *tetB*), transposase coding genes [e.g., *tnXo19* (Tn3 family), and *iSDku1* (IS256 family)] (Figures 2A–D,F). Moreover, four *hin* genes were linked with *tnXo19*, indicating a high transfer of the *hin* gene by transposase-directed horizontal gene transfer (HGT) events, which would also help the HD0315_2 strain to evade host immune responses. Meanwhile, *mccF, macB, entS, alba*, and *bcrA* (coding microcin C7 self-immunity protein, macrolide export ATP-binding/permease protein, enterobactin exporter, antilisterial bacteriocin subtilosin biosynthesis protein, and bacitracin transport ATP-binding protein, respectively) might help the HD0315_2 strain to resist microcin produced from *Paraclostridium* members or microbial toxins from other bacteria. Consequently, these plasmid features would make this strain not only resistant to host responses but also to harsh environments. Hence, *P. bifermentans* HD0315_2 might live under antimicrobial or oxidative stress and might infect the host cell *via* a *Listeria* LIPI-1-like cycle. Thus, the chromosome or plasmid-encoded functions would help *P. bifermentans* HD0315_2 survive during the whole infection cycle by evading host immune responses or resisting medical compounds.

## Conclusion

In summary, we reported the complete genome sequence of *P. bifermentans* HD0315_2 and analyzed its general genomic features, phylogenomic relationship, and pathogenic potential. The results revealed that multiple plasmids are present in *P. bifermentans* strains. Additionally, some species previously identified as non-*P. bifermentans* were reclassified as *P. bifermentans* by the phylogenomic analysis. The pathogenicity proteins encoded by the HD0315_2 strain genome suggested that it can infect host cells *via* a *Listeria* LIPI-1-like cycle. Moreover, both the chromosome and plasmids were found coding abundant antimicrobial or oxidative stress resistance functions. Additionally, transposase-directed HGT generated the distribution of multiple copies of the *hin* gene in the plasmids, which might help the strain evade host immune responses. Therefore, in the present study, we expanded the knowledge regarding the general genomic features of *P. bifermentans*, revealed some of its environmental/host adaptation mechanisms, and proposed, for the first time, its pathogenicity model at the genomic level.

## Data availability statement

The datasets presented in this study can be found in online repositories. The names of the repository/repositories and accession number(s) can be found in the article/Supplementary material.

## Ethics statement

This study was approved by our Institutional Review Board (July 24, 2019; reference No. K-2019-146-01) and was compliant with all relevant ethical regulations. The patients/participants provided their written informed consent to participate in this study. Written informed consent was obtained from the individual(s) for the publication of any potentially identifiable images or data included in this article.

## Author contributions

HZ performed the bacteria isolation and taxa identification. YP and XC performed the WGS-based analysis. HZ, JW, and XC drafted the manuscript. YP, YL, and WH reviewed and revised the manuscript. HH and YN designed the whole study. All authors made substantial and direct contributions to the work, and read and approved the final version of the manuscript.

## Funding

This work was supported by the National Natural Science Foundation of China (81871905 and 41907214); Natural Science Foundation of Guangdong Province (2020A1515011000); and Guangzhou Planned Project of Science and Technology (202002030288 and 202002020012).

## Conflict of interest

The authors declare that the research was conducted in the absence of any commercial or financial relationships that could be construed as a potential conflict of interest.

## Publisher's note

All claims expressed in this article are solely those of the authors and do not necessarily represent those of their affiliated organizations, or those of the publisher, the editors and the reviewers. Any product that may be evaluated in this article, or claim that may be made by its manufacturer, is not guaranteed or endorsed by the publisher.
