# Supplementary material for: Genomic insights from Paraclostridium bifermentans HD0315_2: General features and pathogenic potential
Source: Front Microbiol. 2022 Aug 24;13:928153. doi: 10.3389/fmicb.2022.928153 (PMC9449513; doi:10.3389/fmicb.2022.928153)
Supplement: Supplementary file 2 [file Data_Sheet_2.docx]

**
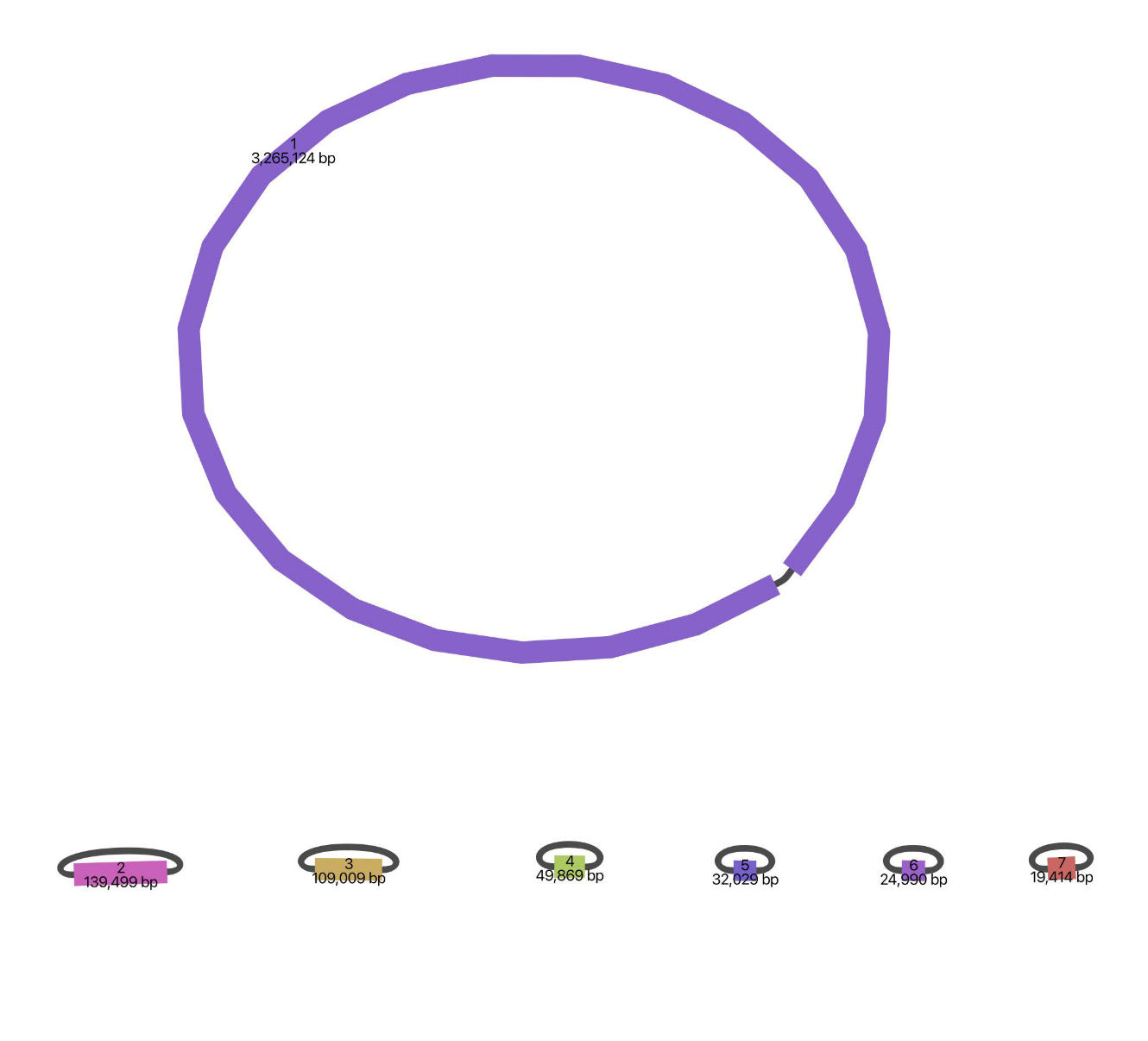
**

Figure S1. Assembly graph generated using Bandage. Seven contigs were circularized with no suspicious connections.


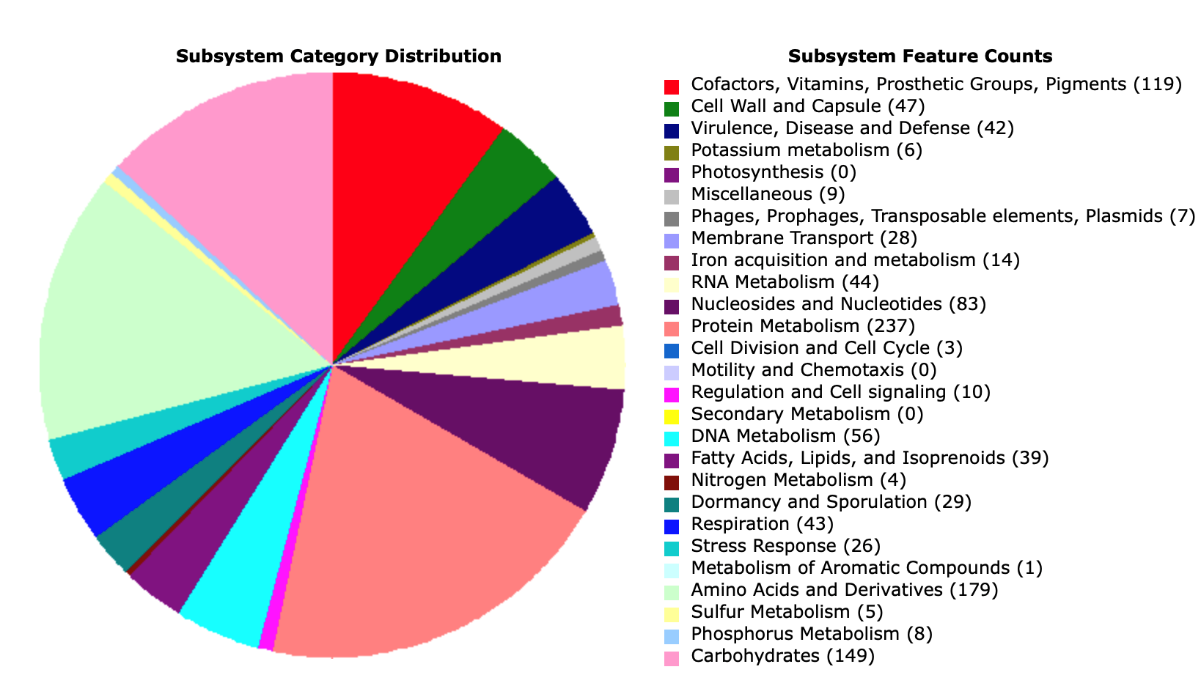


Figure S2. The genomic coded genes annotated to SEED subsystem functions.


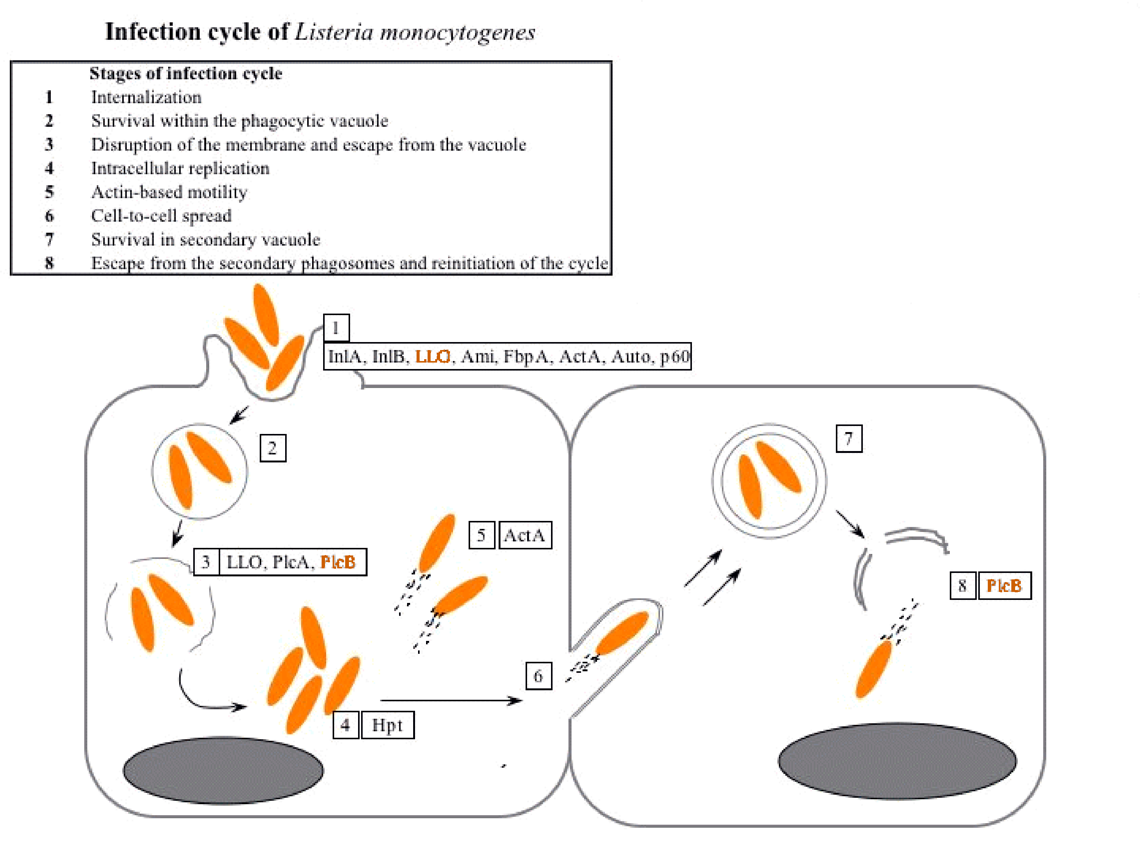


Figure S3. Scheme of the infection cycle of *Listeria* *monocytogenes*.
